# Supplementary material for: Human-driven greenhouse gas and aerosol emissions cause distinct regional impacts on extreme fire weather
Source: Nat Commun. 2021 Jan 11;12:212. doi: 10.1038/s41467-020-20570-w (PMC7801713; doi:10.1038/s41467-020-20570-w)
Supplement: Supplementary file 2 — Supplementary Information [file 41467_2020_20570_MOESM2_ESM.pdf]

Supplementary information for

# Human-driven greenhouse gas and aerosol emissions cause distinct regional impacts on extreme fire weather

Danielle Touma, Samantha Stevenson, Flavio Lehner, Sloan Coats

**Supplementary Table 1:** Models in the fifth phase of the Coupled Model Intercomparison Project (CMIP5) used in Supplementary Figure 12.

| Model | Ensemble member |
|---|---|
| Beijing Normal University Earth System Model (BNU-ESM) | r1i1p1 |
| Canadian Earth System Model v2 (CanESM2) | r2i1p1 |
| Centre National de Recherches Météorologique Climate Model (CNRM-CM5) | r1i1p1 |
| Commonwealth Scientific and Industrial Research Organisation Mk3 Model (CSIRO-Mk3-6-0) | r1i1p1 |
| Geophysical Fluid Dynamics Laboratory Climate Model v3 (GFDL-CM3) | r1i1p1 |
| Geophysical Fluid Dynamics Laboratory Earth System Model (GFDL-ESM) 2G | r1i1p1 |
| Geophysical Fluid Dynamics Laboratory Earth System Model (GFDL-ESM) 2M | r1i1p1 |
| Goddard Institute for Space Studies (GISS) E2-H Model | r6i1p1 |
| Goddard Institute for Space Studies (GISS) E2-R Model | r6i1p1 |
| Institut Pierre Simon Laplace Model (IPSL) CM5A-LR | r2i1p1 |
| Institut Pierre Simon Laplace Model (IPSL) CM5A-MR | r1i1p1 |
| Institut Pierre Simon Laplace Model (IPSL) CM5B-LR | r1i1p1 |

**Supplementary Table 2:** Regions and latitude/longitude boundaries used for regional summaries (shown in Figures 1 and 2).

| Regions | Abbreviation | Latitude boundaries | Longitude boundaries |
|---|---|---|---|
| Western North America | WNA | 30 N - 45 N | 125 W - 115.5 W |
| Eastern North America | ENA | 30 N - 40 N | 89 W - 75 W |
| Amazon | AMZ | 17.5 S - 2.5 N | 75 W - 34.5 W |
| Mediterranean | MED | 36 N - 47 N | 10.5 W - 48 E |
| Equatorial Africa | EQF | 5 N - 17.5 N | 17 W - 40 E |
| Southern Africa | SAF | 28 S - 10 S | 10 E - 50 E |
| Southeast Asia | SEA | 10 S - 35 N | 95 E - 127 E |
| Australia | AUS | 40 S - 11 S | 112 E - 153 E |

## Supplementary Figure 1

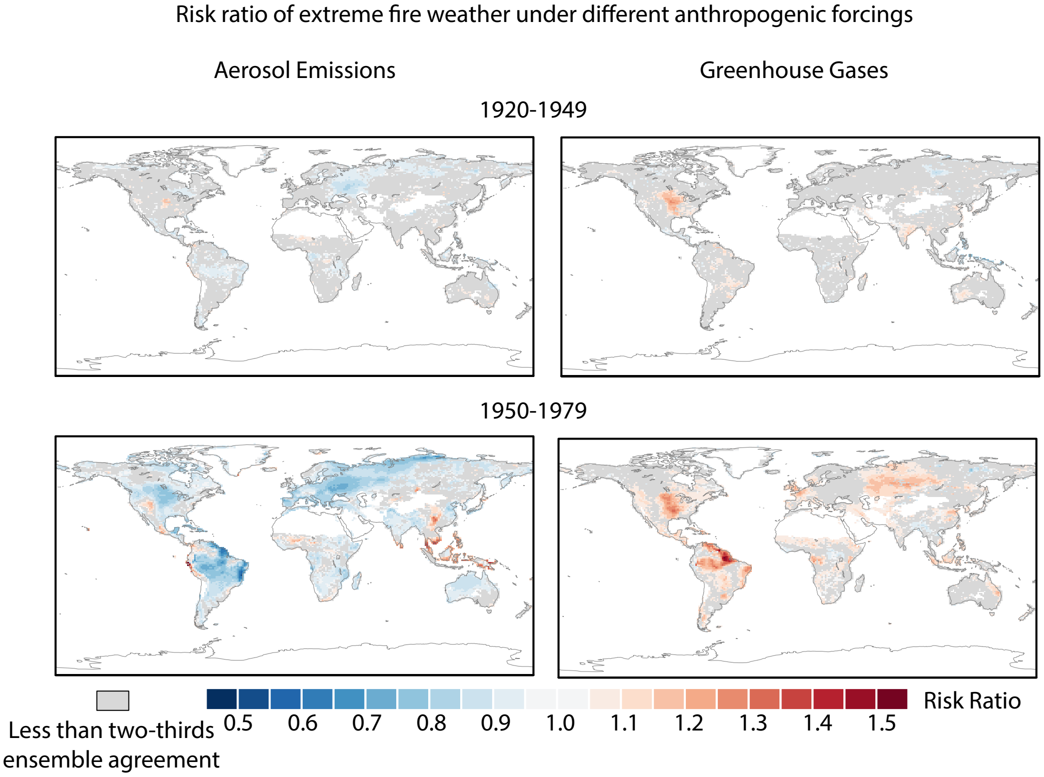


**Anthropogenic impact on extreme fire weather.** Risk ratio (RR) of extreme fire weather under aerosol emissions and greenhouse gases for 1920-1949 and 1950-1979. Grey areas show less than two-thirds ensemble agreement on whether the RR is greater or less than one. Oceans, glaciers, and bare land are masked in white.

## Supplementary Figure 2

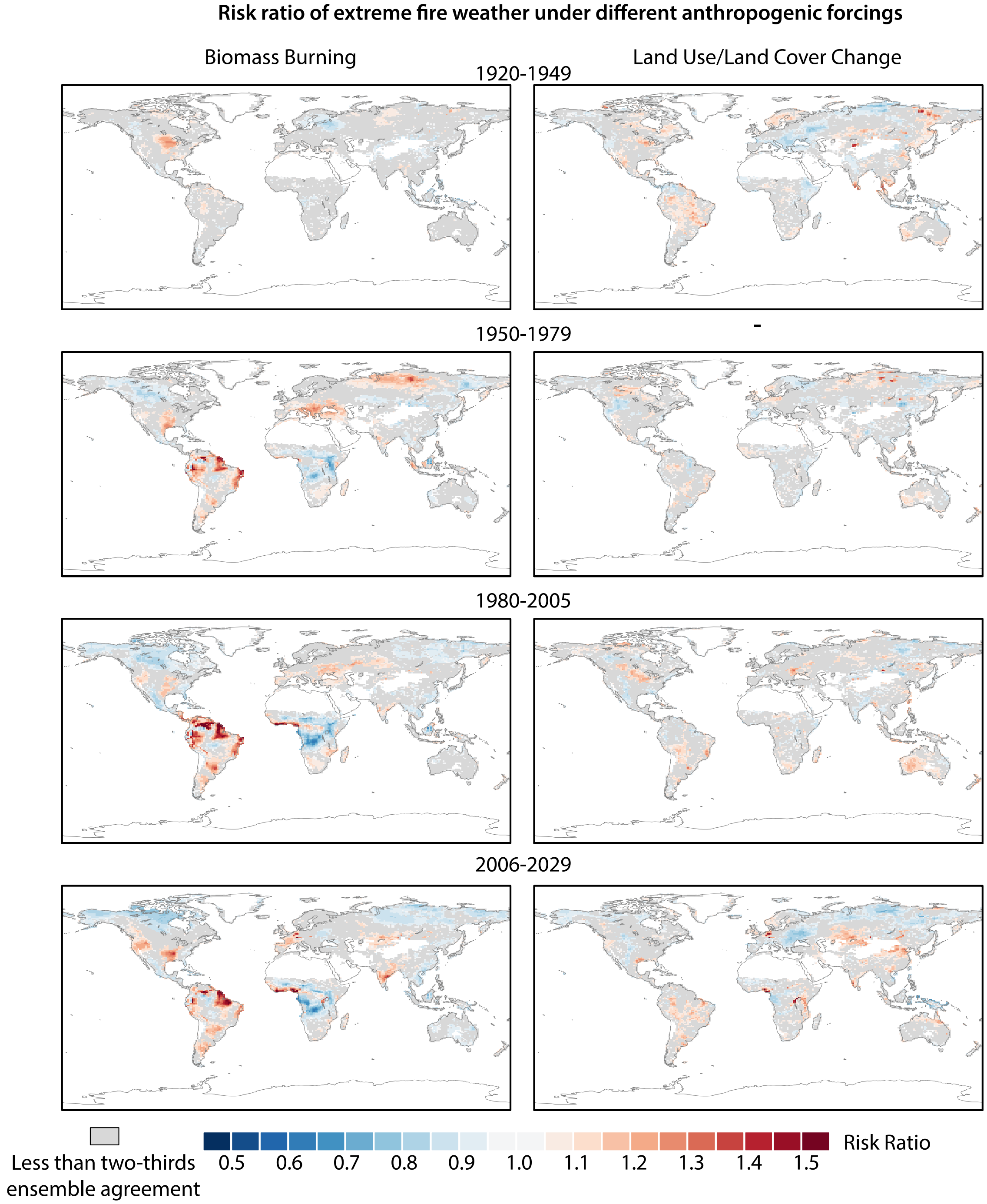


**Anthropogenic impact on extreme fire weather**. Risk ratio (RR) of extreme fire weather under biomass burning and land use land cover change for 1920-1949, 1950-1979, 1980-2005 and 2006-2029. Grey areas show less than two-thirds ensemble agreement on whether the RR is greater or less than one. Oceans, glaciers, and bare land are masked in white.

**Supplementary Figure 3**

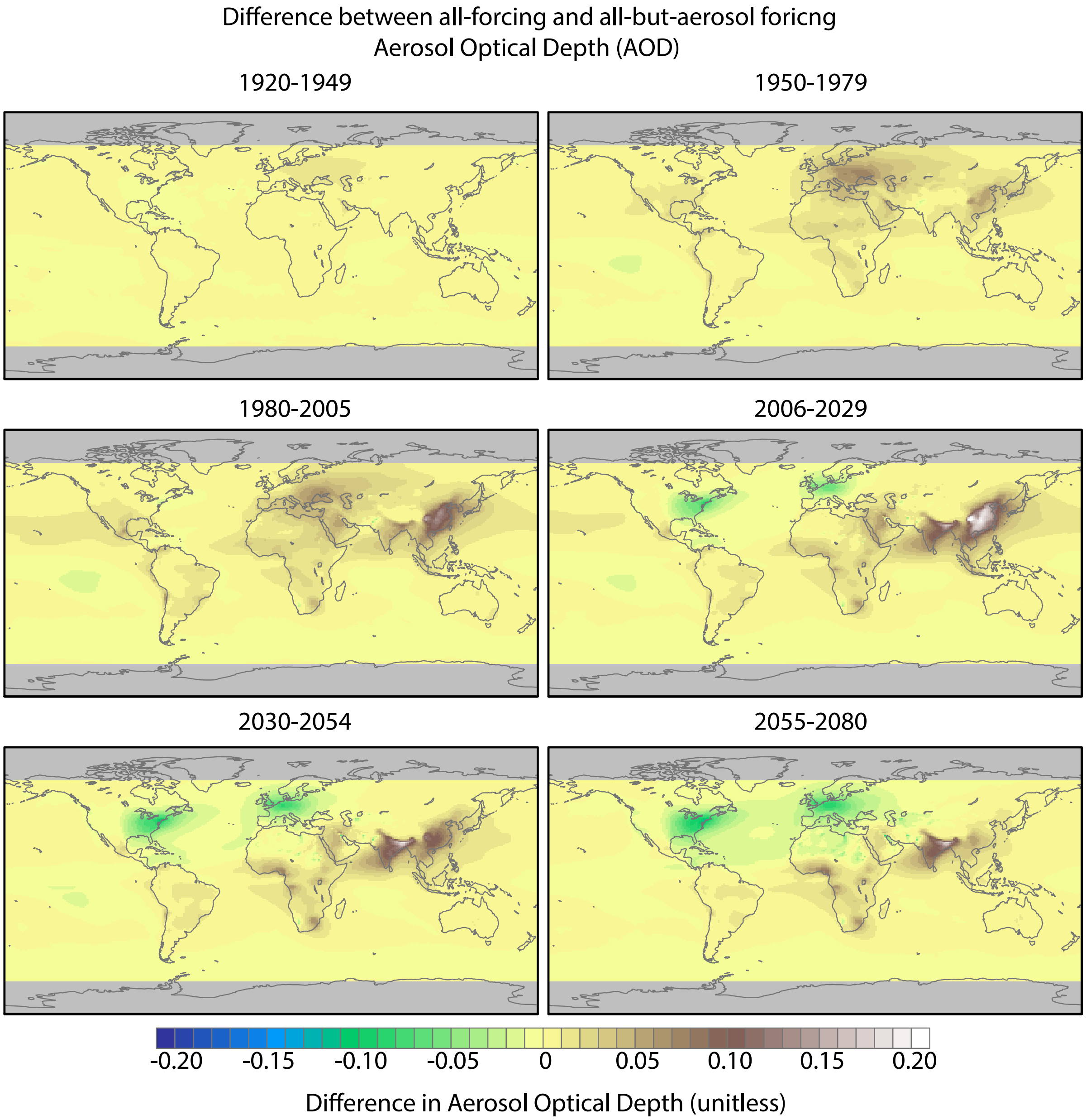


**Changes in aerosol optical depth.** Effect of industrial aerosol emissions (AER) on aerosol optical depth (AOD) for 1920-1949, 1950-1979, 1980-2005, 2006-2029, 2030-2054, and 2055-2080. The shading shows the difference of AOD between the all and the all-but-aerosol forcing ensemble-means averaged over each period. AOD values in polar regions are masked for plotting purposes.

## Supplementary Figure 4

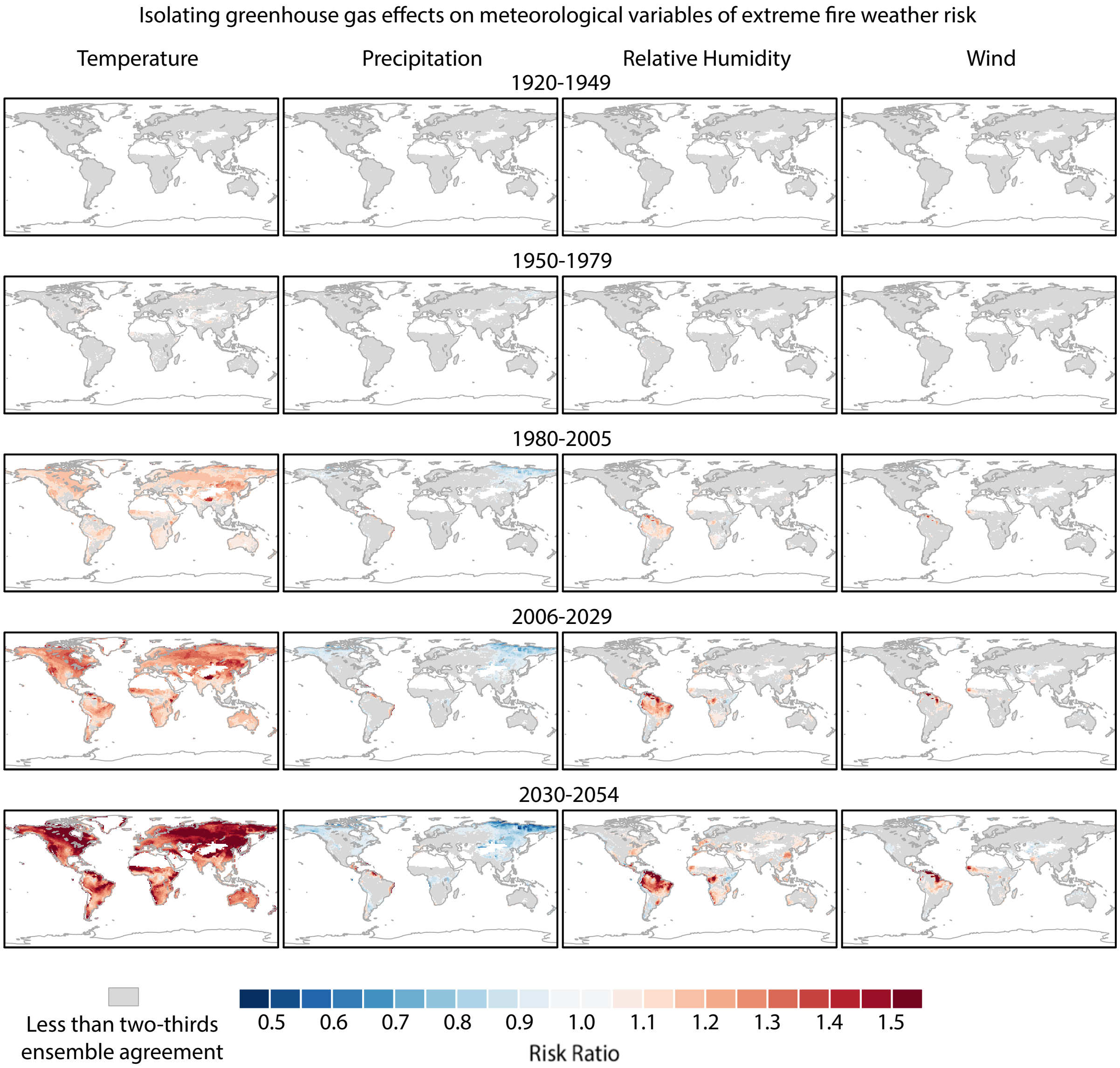


**Isolating the effects of meteorological variables on extreme fire weather risk.** Effect of greenhouse gas (GHG) forcing on extreme wire weather risk due to changes in maximum temperature, precipitation, relative humidity, and windspeed for 1920-1949, 1950-1979, 1980-2005, 2006-2029 and 2030-2054. The risk ratio (RR) is the probability of exceeding the 95$^{th}$ percentile of the baseline daily fire weather index (FWI) distribution in the all-forcing (ALL) ensemble divided by the probability of exceeding that same threshold in the all-forcing ensemble after removing the GHG effect on each variable. Grid points masked in grey have less than two-thirds ensemble agreement on whether the RR is greater or less than one. Oceans, glaciers, and bare land are masked in white.

**Supplementary Figure 5**

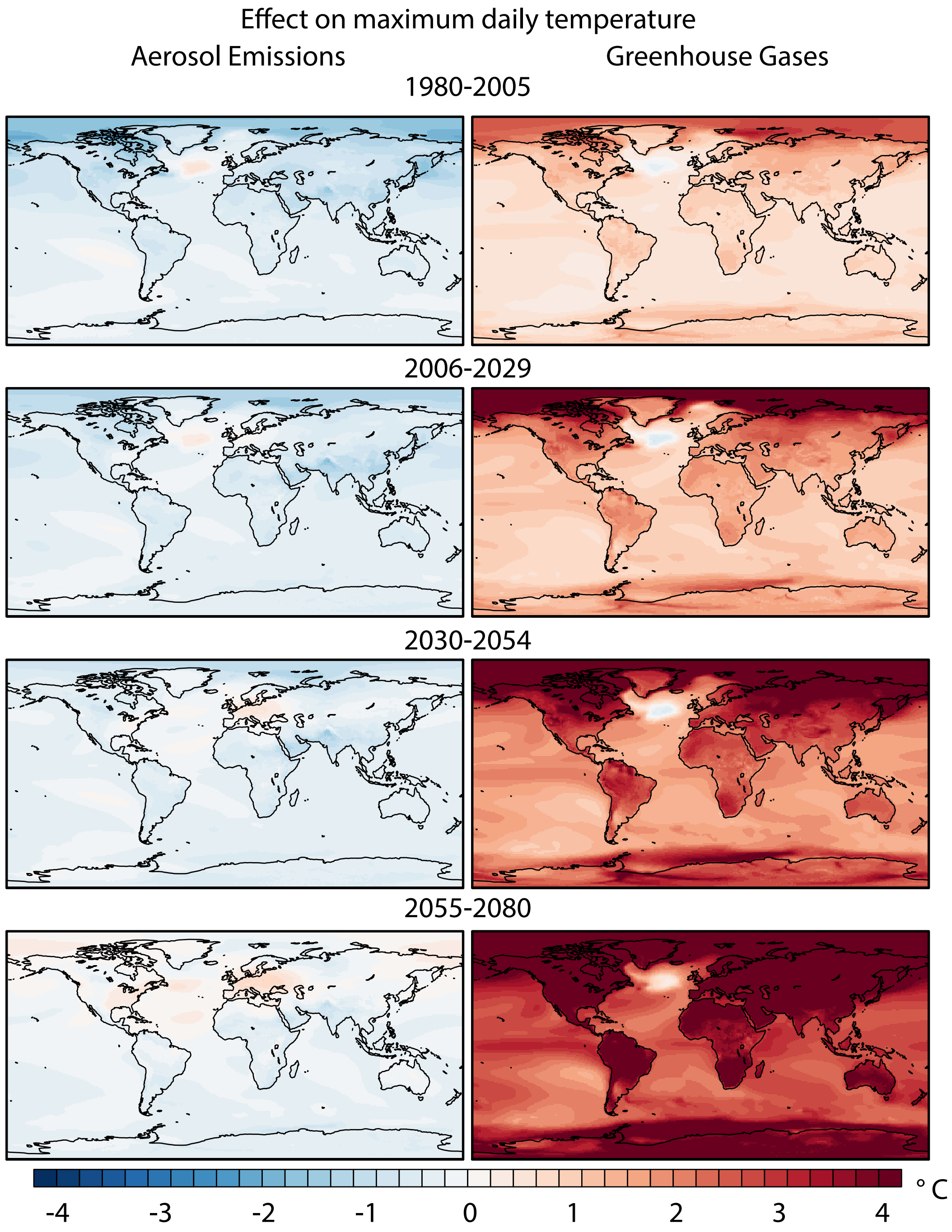


**Anthropogenic effect on maximum daily temperature.** Effect of aerosols (AER) and greenhouse gases (GHG) on maximum daily temperature (°C) for 1980-2005, 2006-2029, 2030-2054, and 2055-2080. The shading shows the difference between the all and the all-but-AER (all-but-GHG) ensemble-means averaged over each period.

**Supplementary Figure 6**

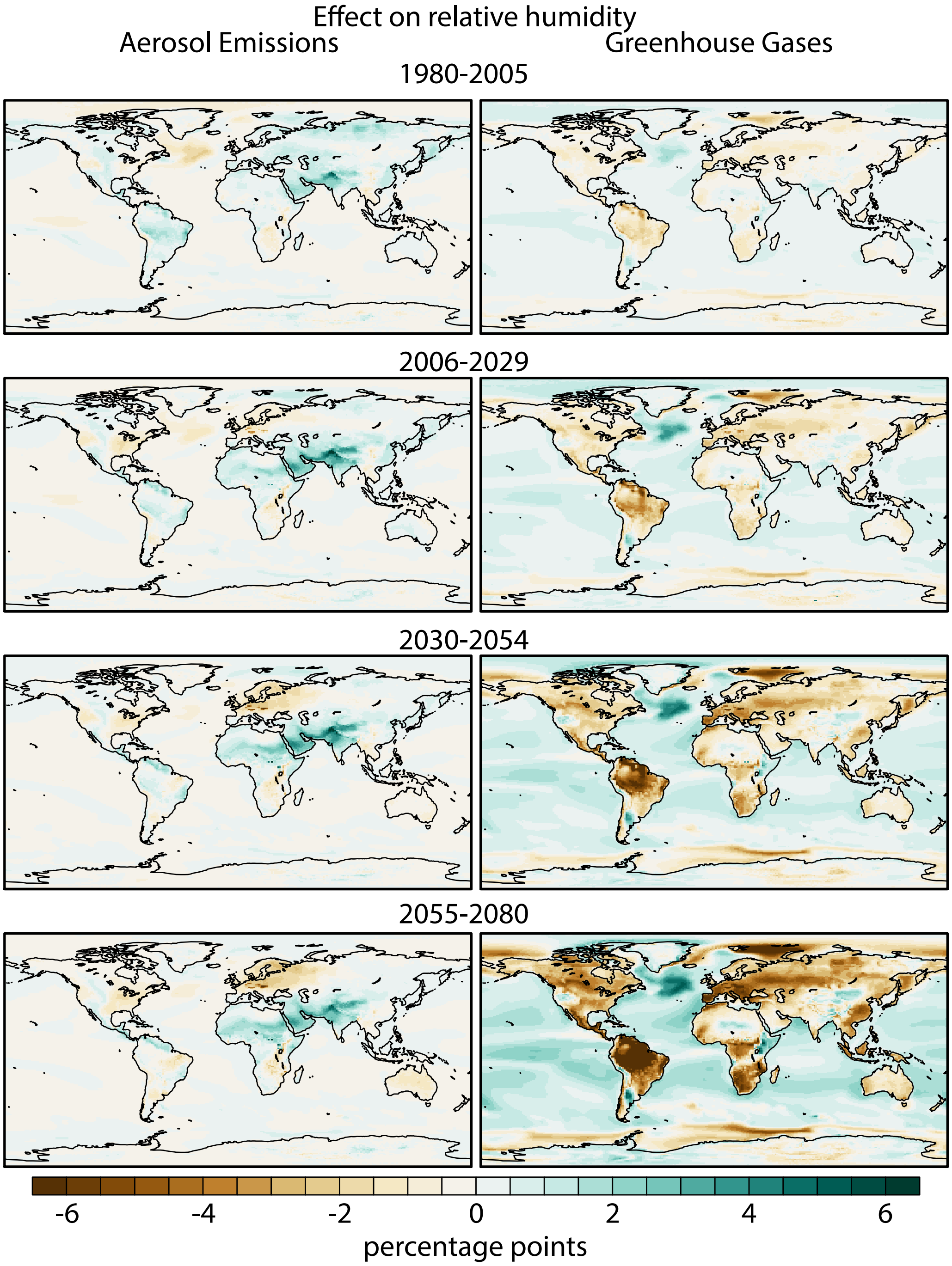


**Anthropogenic impact on relative humidity.** Effect of aerosols (AER) and greenhouse gases (GHG) on relative humidity (percentage points) for 1980-2005, 2006-2029, 2030-2054, and 2055-2080. The shading shows the difference between the all and the all-but-AER (all-but-GHG) ensemble-means averaged over each period.

## Supplementary Figure 7

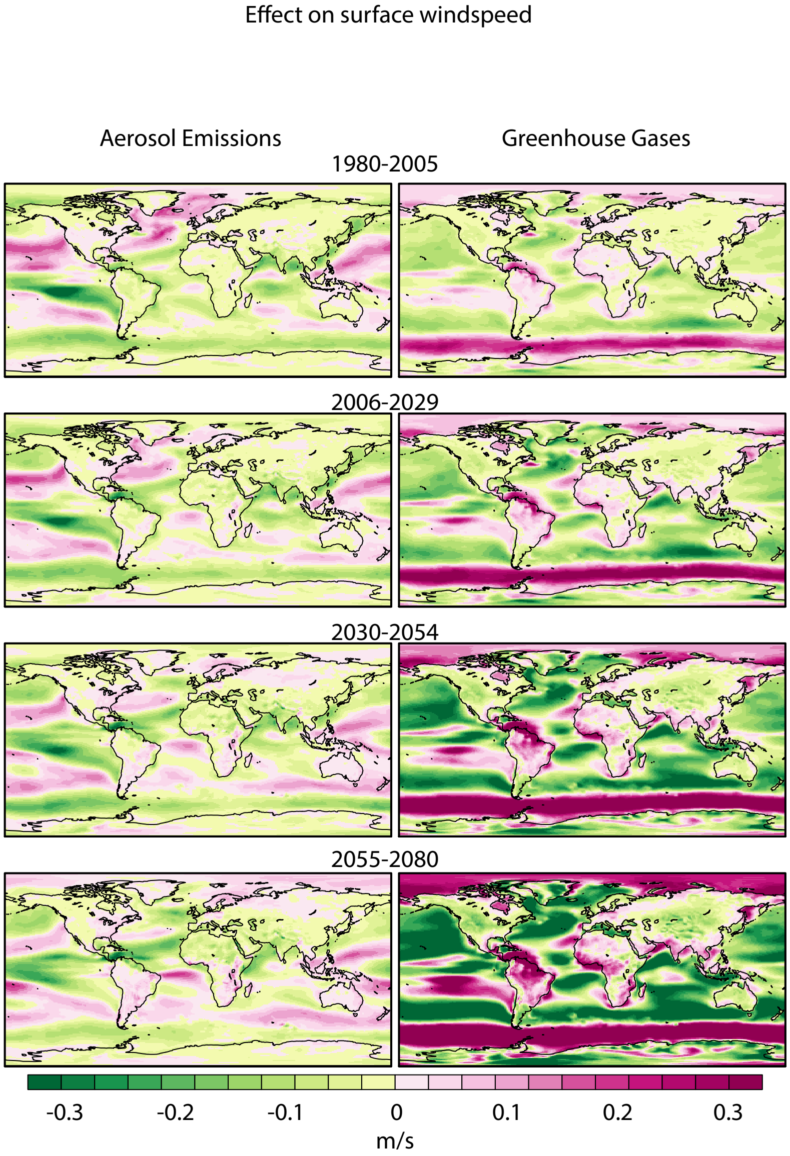


**Anthropogenic effects on surface wind speed.** Effect of aerosols (AER) and greenhouse gases (GHG) on wind speed (m/s) for 1980-2005, 2006-2029, 2030-2054, and 2055-2080. The shading shows the difference between the all and the all-but-AER (all-but-GHG) ensemble-means averaged over each period.

## Supplementary Figure 8

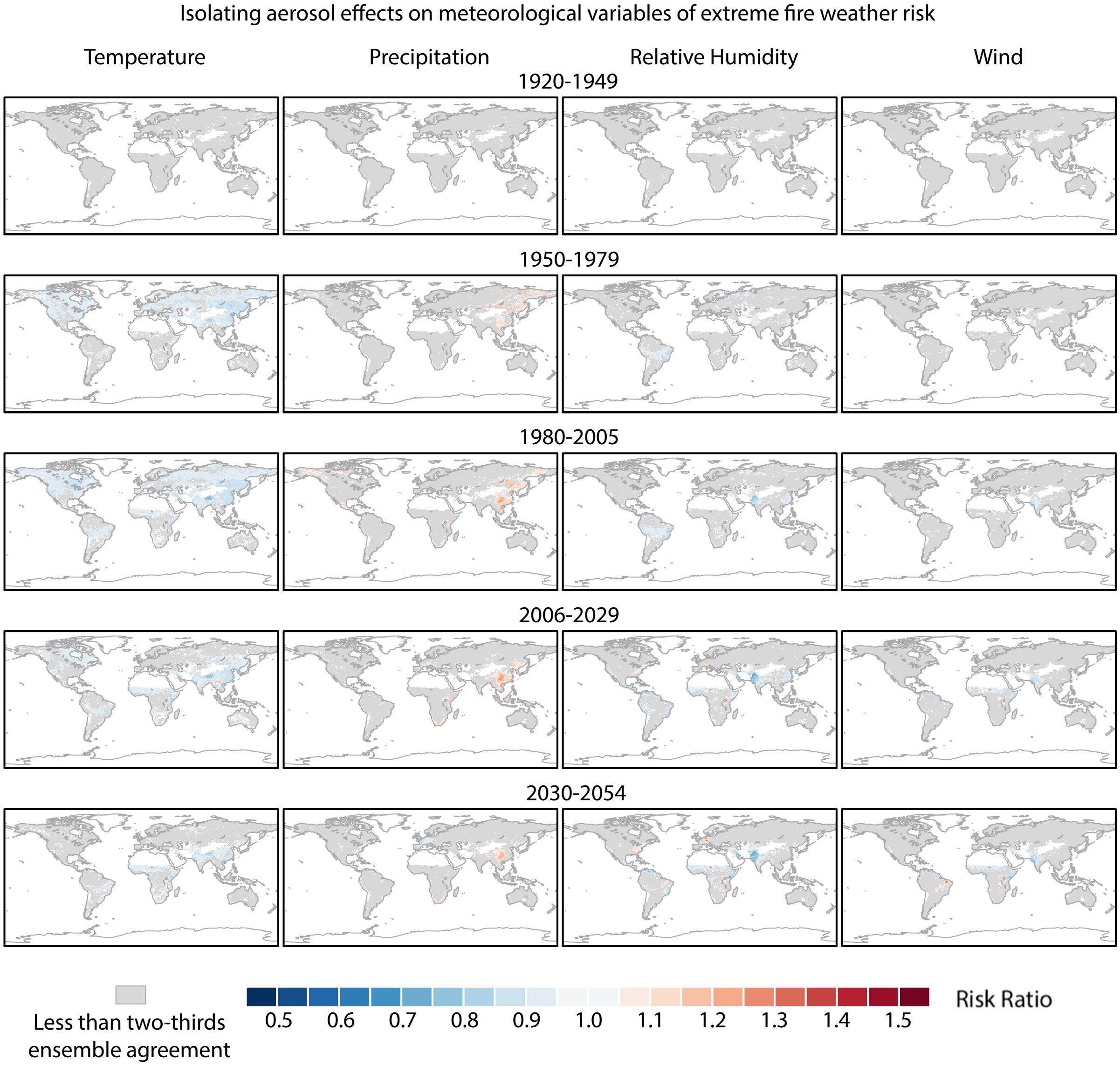


**Isolated effects of meteorological variables on extreme fire weather risk.** Aerosol forcing impacts on extreme fire weather risk due to changes in maximum temperature, precipitation, relative humidity and wind for 1920-1949, 1950-1979, 1980-2005, 2006-2029, and 2030-2054. The risk ratio (RR) is the probability of exceeding the 95$^{th}$ percentile of the baseline daily fire weather index (FWI) distribution in the all-forcing ensemble divided by the probability of exceeding that same threshold in the all-forcing ensemble after removing the aerosol effect on each variable. Grid points masked in grey have less than two-thirds ensemble agreement on whether the RR is greater or less than one. Oceans, glaciers, and bare land are masked in white.

**Supplementary Figure 9**

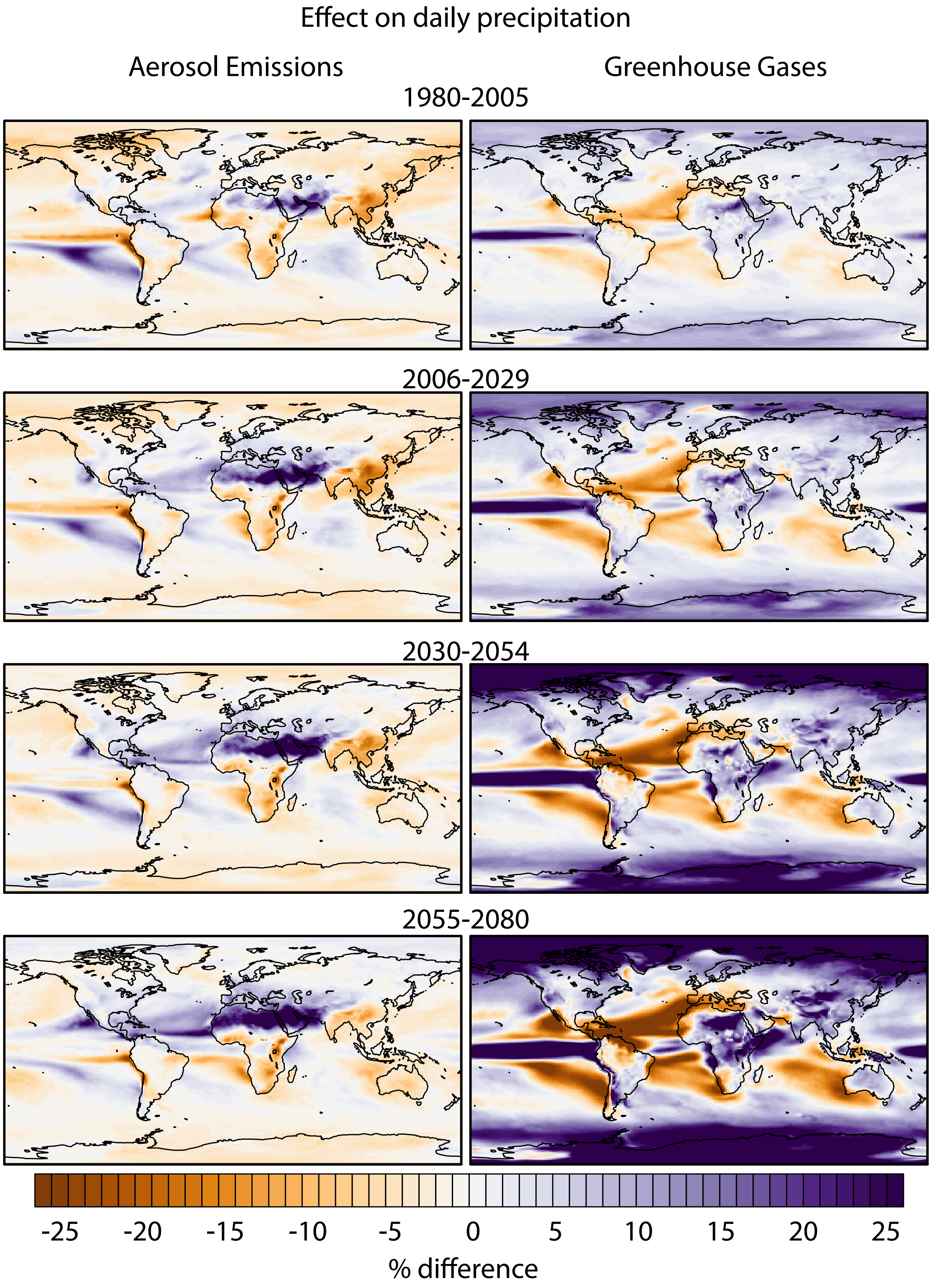


**Anthropogenic effects on daily precipitation.** Effect of aerosols (AER) and greenhouse gases (GHG) on daily precipitation (%) for 1980-2005, 2006-2029, 2030-2054, and 2055-2080. The shading shows the percent difference between the all-forcing and the all-but-AER (all-but-GHG) ensemble-means averaged over each period.

## Supplementary Figure 10

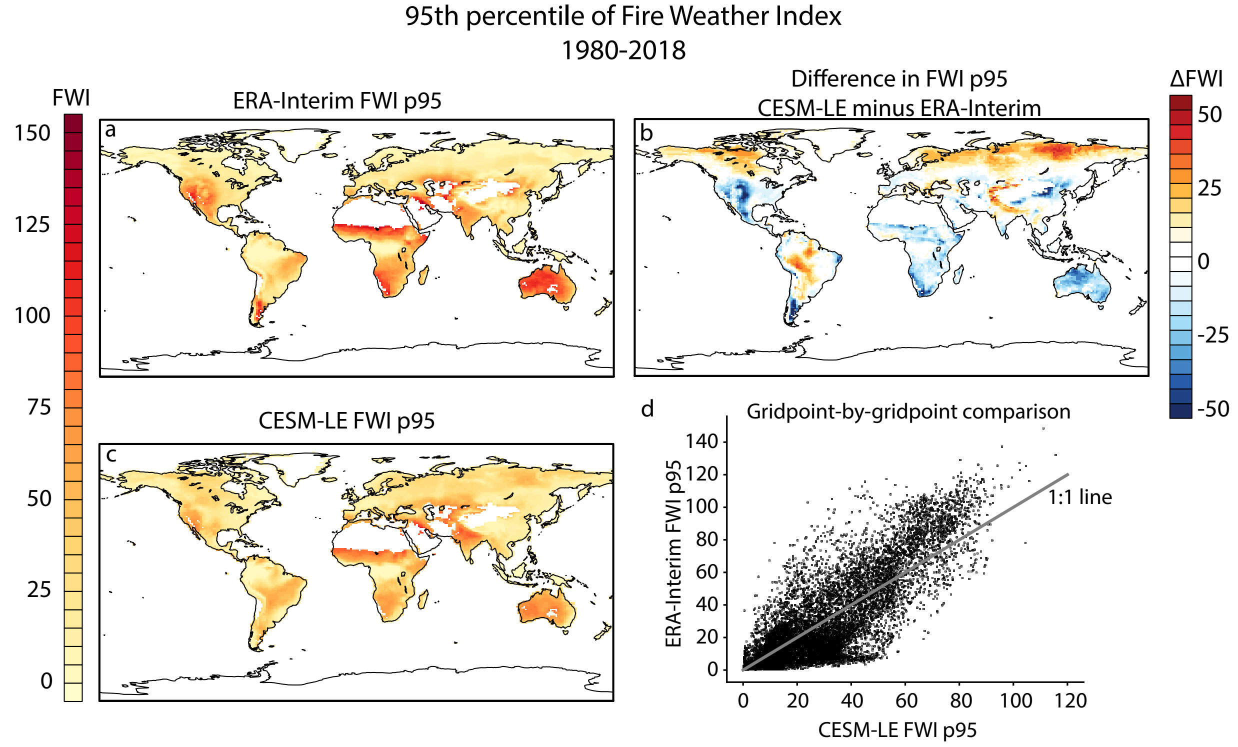


**Comparison between CESM Large Ensemble and ERA-Interim.** 95th percentile of the daily fire weather index (FWI) for ERA-Interim (a) and CESM-LE (c) for 1980-2018, and their difference (b). d) Gridpoint-by-gridpoint comparison for unmasked locations of the 95$^{th}$ percentile of the daily FWI for ERA-Interim and CESM-LE.

## Supplementary Figure 11

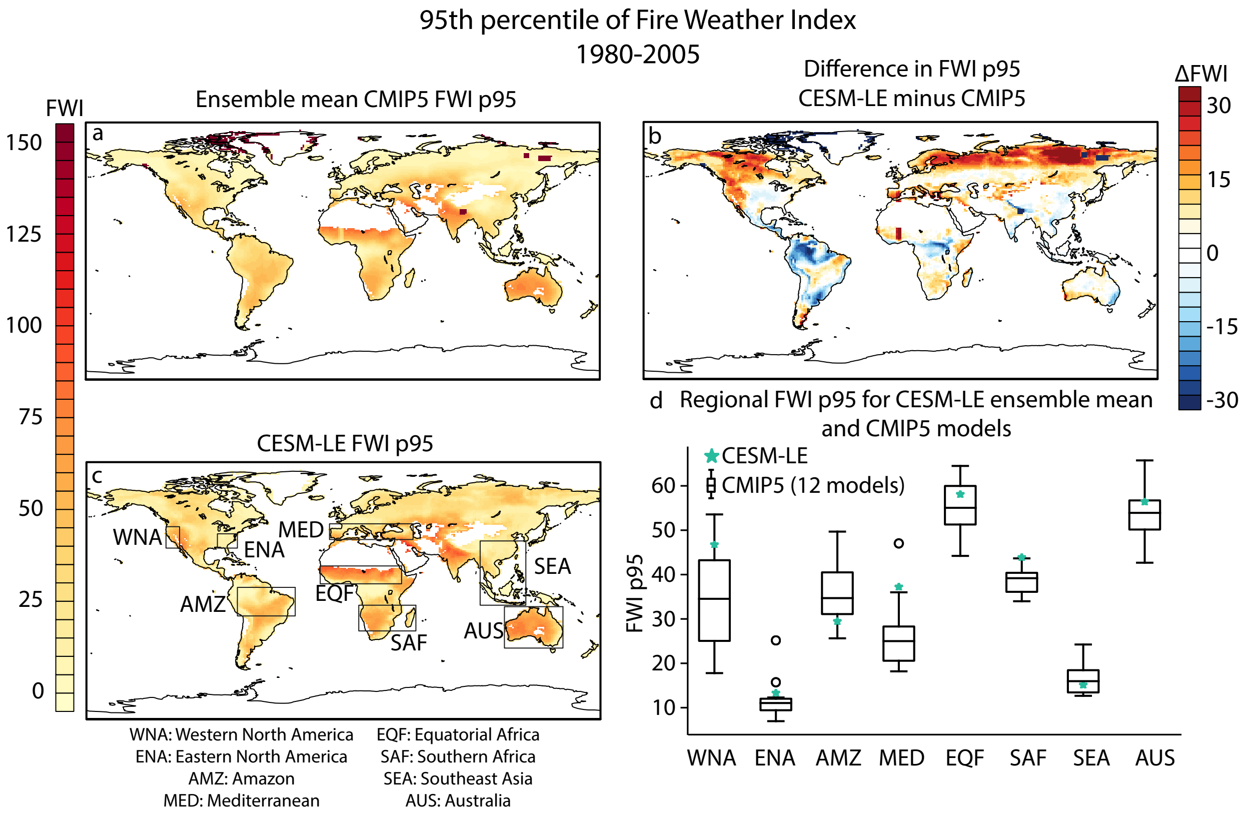


**Comparison between CESM Large Ensemble and CMIP5 models.** Model-mean of the 95th percentile of the daily fire weather index (FWI) for CMIP5 (a) and CESM-LE (c) for 1980-2005, and their difference (b). d) Range of CMIP5 models FWI p95 (boxplot) and CESM-LE FWI p95 (star) for each region. Region boundaries are shown in (c) and in Supplementary Table 2.

**Supplementary Figure 12**

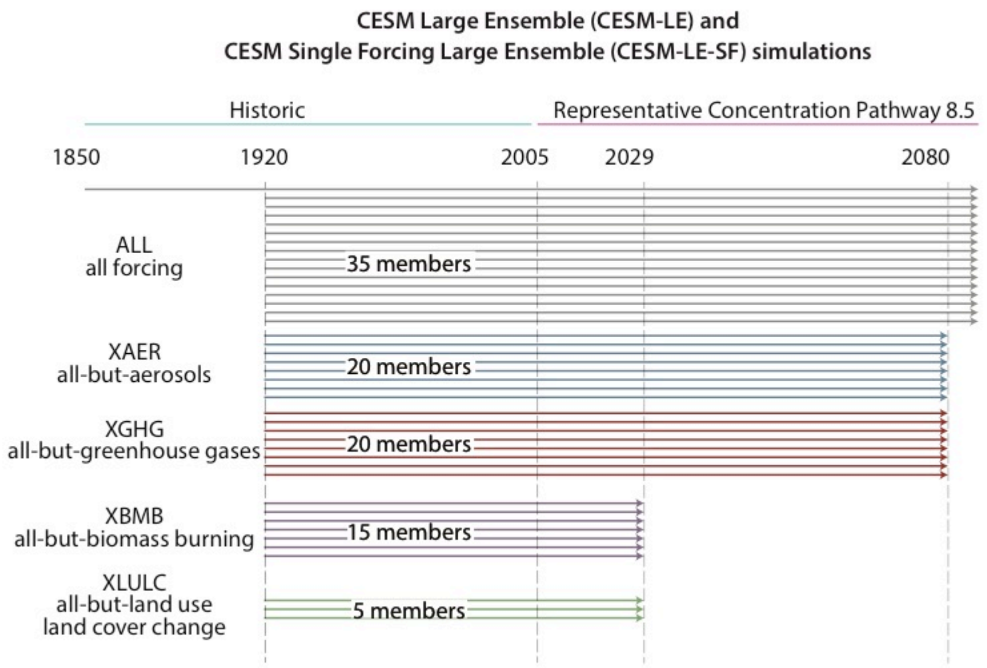


**Schematic describing the CESM Large Ensemble (CESM-LE) and Single Forcing Large Ensemble (CESM-LE-SF) experiments.** The all forcing experiment (ALL) has 35 members spanning from 1920-2100 (the first member starts on 1850). The all-but-aerosol (XAER) and all-but-greenhouse gas (XGHG) experiments have 20 members each and span from 1920-2080. The all-but-biomass burning (XBMB) and all-but-land use land cover change (XLULC) have 15 and 5 members, respectively, and span from 1920-2030. Historic forcings are used from 1920-2005, and Representative Concentration Pathway 8.5 forcings are used from 2005-2100 in all experiments.
